# Examining the Safety Profile of Janus Kinase (JAK) Inhibitors in the Management of Immune-Mediated Diseases: A Comprehensive Review

**DOI:** 10.3390/life13122244

**Published:** 2023-11-22

**Authors:** Krasimir Kraev, Mariela Gencheva Geneva-Popova, Bozhidar Krasimirov Hristov, Petar Angelov Uchikov, Stanislava Dimitrova Belova-Popova, Maria Ilieva Kraeva, Yordanka Mincheva Basheva-Kraeva, Nina Staneva Stoyanova, Vesela Todorova Mitkova-Hristova, Maria Stoyanova Koleva-Ivanova, Daniela Ivova Taneva, Atanas Slavchev Ivanov

**Affiliations:** 1 Department of Propedeutics of Internal Diseases, Medical Faculty, Medical University of Plovdiv, 4000 Plovdiv, Bulgaria; 2 Second Department of Internal Diseases, Medical Faculty, Medical University of Plovdiv, 6000 Plovdiv, Bulgaria; 3 Department of Special Surgery, Medical Faculty, Medical University of Plovdiv, 6000 Plovdiv, Bulgaria; 4 Department of Otorhynolaryngology, Medical Faculty, Medical University of Plovdiv, 6000 Plovdiv, Bulgaria; 5 Department of Ophthalmology, Faculty of Medicine, Medical University of Plovdiv, University Eye Clinic, University Hospital, 4000 Plovdiv, Bulgaria; 6 Department of General and Clinical Pathology, Faculty of Medicine, Medical University of Plovdiv, 4000 Plovdiv, Bulgaria; 7 Department of Nursing Care, Faculty of Public Health, Medical University of Plovdiv, 4000 Plovdiv, Bulgaria; 8 Department of Urology and General Medicine, Medical University of Plovdiv, 4000 Plovdiv, Bulgaria

**Keywords:** JAK inhibitors, immune-mediated diseases, safety profile, adverse events

## Abstract

Janus kinase (JAK) inhibitors have heralded a paradigm shift in the management of immune-mediated diseases. While their efficacy is well-established, the safety concerns associated with these agents, particularly regarding thromboembolic events (TE), remain a focus of extensive research and clinical scrutiny. This comprehensive literature review embarks on an exploration of the multifaceted landscape of JAK inhibitors, providing insights into their safety profiles across diverse immune-mediated diseases. The introduction highlights the transformative influence of JAK inhibitors in the treatment of immune-mediated diseases. Historically, the therapeutic arsenal for these conditions included corticosteroids, disease-modifying antirheumatic drugs (DMARDs), and biologics. The advent of JAK inhibitors has revolutionized this landscape, although concerns about their safety persist. This review strives to comprehensively evaluate their safety, amalgamating knowledge from multiple studies and trials. The subsequent sections delve into the safety of specific JAK inhibitors in the context of rheumatoid arthritis, inflammatory bowel diseases, and dermatologic conditions and their associations with venous thromboembolism. The evolving understanding of TE risk, particularly the intricate relationship between these agents and immune-mediated diseases, is meticulously unravelled. The concluding remarks underscore the dynamic nature of TE risk assessment with regard to immune-mediated diseases involving JAK inhibitors. It underscores that risk assessment is multifactorial, influenced not only by the choice of JAK inhibitor but also by the nuances of the underlying immune-mediated disease and the unique patient characteristics. This review offers a holistic perspective on TE risks associated with JAK inhibitors and contributes to the ongoing dialogue regarding their safety in the realm of immune-mediated diseases.

## 1. Introduction

Immune-mediated diseases encompass a wide range of conditions, each marked by a dysregulated immune system, causing inflammation and damage to various organs and tissues. Traditionally, the therapeutic options for these conditions included corticosteroids, disease-modifying antirheumatic drugs (DMARDs), and biologic agents [1,2]. However, the introduction of Janus kinase (JAK) inhibitors has brought about a significant shift in the treatment landscape, offering new hope to both patients and healthcare providers (Table 1). These diseases, such as rheumatoid arthritis (RA), inflammatory bowel diseases (IBD), psoriasis, psoriatic arthritis (PsA), atopic dermatitis, and alopecia areata (AA), often share a common underlying thread of immune dysfunction leading to chronic inflammation and adverse clinical outcomes. The intricate pathogenesis of these diseases necessitates a diverse range of therapeutic strategies, and the development and clinical use of JAK inhibitors have emerged as a groundbreaking approach to managing immune-mediated diseases [3].

**Figure 1:**
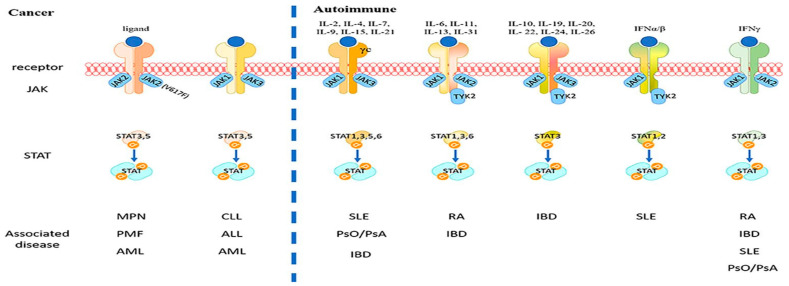
Overview of Janus kinase (JAK) signalling pathways in autoimmune diseases and cancers. The binding of different type I and II cytokines to specific receptor subunits associated with JAKs leads to the activation of specific downstream intracellular signals. STATs represent a prominent class of molecules that can transmit signals from cytokine receptors to the nucleus to activate the transcription of several specific target genes. Different JAK/STAT signalling pathways contribute to the pathogenesis of various immune-mediated diseases [4]. MPN—myeloproliferative neoplasms, PMF—primary myelofibrosis, AML—acute myeloid leukaemia, CLL—chronic lymphocytic leukaemia, ALL—adult acute lymphoblastic leukaemia, SLE—systemic Lupus erythematosus, PsO—psoriasis, PsA—psoriatic arthritis, IBD—inflammatory bowel diseases, and RA—rheumatoid arthritis.

Cytokines are essential for the pathogenesis of immune-mediated illnesses, which each have distinct cytokine profiles. The rapidly expanding body of research in the field of rheumatic and malignant disorders has demonstrated effective therapeutic outcomes from targeting the JAK/STAT pathway in type I and type II cytokine signal transduction. The pathways of JAK/STAT signalling pathway activation in the development of many diseases are summarized in Figure 1 [4].

**Figure 2:**
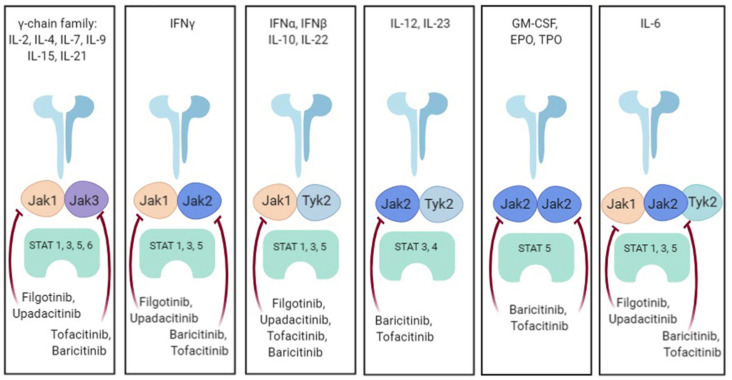
Cytokine signalling through JAK/Stat combination [2].

The JAK family consists of four members: JAK1, JAK2, JAK3, and TYK2. To signal, each cell surface receptor requires a pair of JAKs, either identical homodimers (e.g., JAK2/JAK2) or heterodimers (e.g., JAK1/JAK3). This, in turn, activates STAT proteins (signal transducers and activators of transcription), which target gene promoters to initiate transcription. Each JAK pair has distinct activation ligands and downstream effector functions. Figure 2 illustrates the distinct Janus kinase (JAK) and its corresponding JAK inhibitors [2].

JAK inhibitors, a family of intracellular enzymes that modulate cytokine and growth factor signalling, have shown remarkable therapeutic benefits in clinical trials and real-world settings for a range of immune-mediated diseases. These drugs have exhibited the ability to control symptoms, reduce inflammation, and even facilitate clinical remission in conditions like RA and IBD [5]. Dermatologic conditions like psoriasis, AA, and atopic dermatitis have also witnessed the potential of JAK inhibitors in targeting underlying inflammatory pathways.

However, as the use of JAK inhibitors continues to expand, concerns about their safety profiles persist. Safety is a paramount consideration in healthcare, requiring a balance between the potential benefits and risks of these innovative medications. Some primary safety concerns associated with JAK inhibitors include the risk of serious infections, malignancies, venous thromboembolism, herpes zoster infection, and laboratory abnormalities [6,7,8]. This literature review undertakes a comprehensive examination of the safety profiles of JAK inhibitors, specifically tofacitinib, upadacitinib, and baricitinib, in the context of immune-mediated diseases. By synthesising findings from numerous studies and clinical trials, it aims to provide a nuanced understanding of the safety landscape, illuminate potential concerns, and highlight areas requiring further research and vigilance.

The rationale for this in-depth safety assessment of JAK inhibitors is multifaceted. It enables clinicians to make informed treatment decisions, weighing the potential risks against the clinical benefits offered by these drugs. Effective patient counselling and shared decision making between healthcare providers and patients depend on a deep understanding of these medications’ safety profiles. Ongoing research and post-marketing surveillance are necessary to monitor the long-term safety of JAK inhibitors continuously. The emergence of new data can provide valuable insights into previously unanticipated adverse events or long-term effects. With the expanding use of JAK inhibitors across various immune-mediated diseases, these medications have become a focal point of safety monitoring in real-world clinical practice. This review contributes to our growing understanding of the safety of JAK inhibitors in real-world clinical practice, providing clinicians with the knowledge required to make informed treatment decisions while prioritising the well-being of patients living with immune-mediated diseases.

## 2. The Safety of Tofacitinib, Upadacitinib, Filgotinib, and Baricitinib for Patients with Rheumatoid Arthritis

Rheumatoid arthritis (RA) is a chronic autoimmune disease characterized by inflammation of the synovium, leading to joint damage and disability if not effectively managed. The introduction of Janus kinase (JAK) inhibitors has marked a significant advancement in the treatment of RA, offering patients an alternative to traditional disease-modifying antirheumatic drugs (DMARDs) and biologics. The tabulated data herein encapsulate the most recent clinical trials extracted from PubMed, meticulously elucidating the efficacy and safety profiles of currently available Janus kinase (JAK) inhibitors (Table 2).

**Figure 3:**
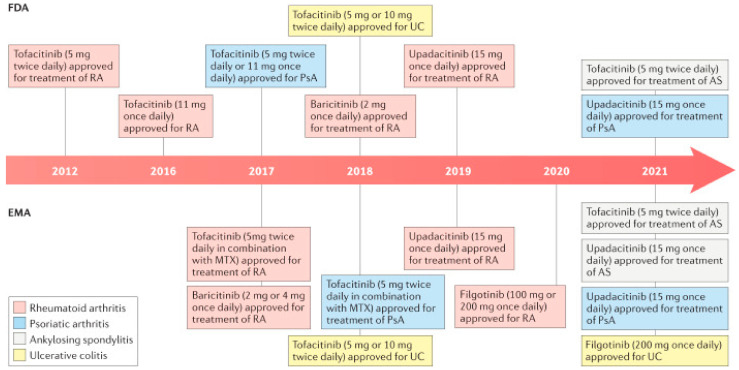
Timeline of approved indications for Janus kinase inhibitors for rheumatic diseases [19].

This section provides a comprehensive exploration of the safety profiles of four JAK inhibitors, namely, tofacitinib, upadacitinib, filgotinib, and baricitinib, in the context of RA (Figure 3).

### 2.1. Tofacitinib

Tofacitinib, the first JAK inhibitor approved for the treatment of RA, has been widely studied, shedding light on its efficacy and safety profile [5,20]. Clinical trials, including the ORAL (Oral Rheumatoid Arthritis Trials) series, have been pivotal in establishing the role of tofacitinib in RA management. The safety findings from these trials and real-world data provide valuable insights [19].

Tofacitinib’s safety profile, like the profiles of other JAK inhibitors, is closely scrutinized for several key aspects. One of the primary concerns is the risk of serious infection. In clinical trials, tofacitinib demonstrated an increased risk of serious infection compared to traditional DMARDs [5,6,7,8,20]. This elevated risk is attributed to its mechanism of action, which includes the inhibition of JAK pathways involved in immune responses. Tofacitinib also raises concerns about malignancy, specifically non-melanoma skin cancer, lymphoma, and lung cancer [8]. While clinical trial data suggested a potential increased risk of malignancies, long-term data are essential to draw concrete conclusions.

Herpes zoster infection is another safety consideration. Studies have indicated an increased risk of herpes zoster when administering tofacitinib, particularly for older patients and those receiving higher doses. This underscores the importance of careful patient selection and monitoring [8].

Laboratory abnormalities, including lipid profile changes and liver enzyme elevations, are also associated with tofacitinib [5,6]. Clinicians should conduct regular monitoring to identify and manage these adverse events promptly.

### 2.2. Upadacitinib

Upadacitinib, a newer JAK inhibitor, has emerged as an effective treatment option for RA. Its clinical development included the SELECT (Safety and Efficacy of Upadacitinib in Patients with Active Rheumatoid Arthritis) trial program, offering insights into its safety profile [21].

The risk of serious infection is a primary focus when assessing upadacitinib’s safety. Clinical trials have reported slightly elevated rates of serious infections, but, overall, the risk appears to be manageable. Notably, upadacitinib has not been associated with an increased risk of tuberculosis, a crucial consideration in countries with a higher prevalence of this infection [22].

The risk of malignancy is a concern, as is the case with other JAK inhibitors. While the data do not indicate a clear association between upadacitinib and malignancies, ongoing vigilance and long-term studies are vital for a more comprehensive assessment [21,22].

Gastrointestinal perforations, uncommon but serious adverse events, have been reported in clinical trials involving upadacitinib. Patients should be educated about the symptoms and signs of these injuries, and healthcare providers should promptly evaluate any suspected cases [21].

The impact on haematological parameters, such as anaemia and neutropenia, is monitored during upadacitinib treatment, requiring regular laboratory assessments. Importantly, the safety and efficacy of upadacitinib in real-world settings are continuously investigated to provide a more complete understanding of its risk–benefit profile [23].

### 2.3. Filgotinib

Filgotinib, another JAK inhibitor, has been assessed for its safety and efficacy in treating RA through clinical trials such as the FINCH (Filgotinib in the Management of Rheumatoid Arthritis) program. This has provided valuable insights into the drug’s safety considerations [24].

As with other JAK inhibitors, the risk of serious infections is a prominent concern. In clinical trials, filgotinib was associated an increased risk of these infections compared to traditional DMARDs. Careful patient selection and monitoring are essential to mitigate this risk [25].

Malignancy risk, including lymphoma, is a focus of safety assessments. While clinical trial data did not indicate a notably increased risk, post-marketing surveillance and long-term studies are necessary for a more comprehensive understanding [25,26].

In the context of cardiovascular events, filgotinib was associated with an increased risk of venous thromboembolism (VTE). However, the VTE risk is notably lower in real-world studies compared to clinical trials, highlighting the importance of studying drugs in diverse patient populations.

Gastrointestinal perforations and hepatotoxicity have also been reported following treatment with filgotinib, underscoring the need for vigilant monitoring and patient education [24,25,26].

### 2.4. Baricitinib

Baricitinib, a JAK inhibitor approved for RA treatment, has undergone extensive evaluation in clinical trials like the RA-BEAM (Rheumatoid Arthritis: Baricitinib in Long-term Experience) and RA-BUILD trials, contributing to our understanding of its safety profile [27].

Serious infections are among the primary safety considerations for baricitinib. Clinical trial data have revealed an association with a slightly increased risk compared to traditional DMARDs, emphasising the importance of ongoing monitoring and infection prevention strategies. The risk of malignancy has also been assessed, with some studies suggesting a potential association with lymphoma. Long-term data are crucial for more definitive conclusions. Gastrointestinal perforations and laboratory abnormalities, including lipid changes and liver enzyme elevations, have been reported after treatment with baricitinib. Clinicians should be vigilant in monitoring patients for these potential adverse events [28].

Furthermore, venous thromboembolism (VTE) risk is notable with baricitinib, albeit more so in clinical trials than in real-world settings. These findings highlight the significance of assessing real-world data for a more accurate representation of a drug’s safety profile [27,28].

In conclusion, while tofacitinib, upadacitinib, filgotinib, and baricitinib offer valuable treatment options for RA, their safety profiles necessitate close monitoring and the consideration of potential risks. The risk of serious infections and malignancies, herpes zoster, gastrointestinal perforations, and laboratory abnormalities are central safety concerns. Real-world studies provide essential insights into the safety of these JAK inhibitors outside the controlled environment of clinical trials. The ongoing collection of long-term data and post-marketing surveillance are paramount in refining our understanding of their safety profiles, ultimately allowing clinicians to make informed decisions regarding RA treatment. As patients continue to benefit from these innovative therapies, maintaining a vigilant focus on their safety is paramount to optimising their risk–benefit balance.

## 3. Safety of JAK Inhibitors in Treating Inflammatory Bowel Diseases (IBD)

Inflammatory Bowel Diseases (IBD), a category of immune-mediated disorders that includes Crohn’s disease and ulcerative colitis, pose significant challenges to patients and healthcare providers alike. The chronic nature of these conditions and their potential to induce severe complications necessitate effective treatment strategies. While conventional therapies and biologics have been staples in managing IBD, the advent of Janus kinase (JAK) inhibitors has introduced a new dimension to IBD management [29].

Patients with IBD often grapple with the debilitating effects of chronic intestinal inflammation, leading to symptoms such as abdominal pain, diarrhoea, and fatigue. The quest for effective treatments led to the evaluation of JAK inhibitors’ safety and efficacy in this context. Tofacitinib, the first JAK inhibitor approved for ulcerative colitis, demonstrated its efficacy in clinical trials. The pivotal Phase 3 OCTAVE trials showcased substantial improvements in the rates of clinical remission and mucosal healing among patients with moderate to severe ulcerative colitis (Sandborn et al., 2017), [30]. This marked a significant milestone in the management of this condition. However, the safety of tofacitinib in IBD patients, particularly with respect to the risk of infections and malignancies, has drawn considerable attention.

In terms of adverse events, patients with IBD receiving tofacitinib were found to exhibit elevated rates of infections in clinical trials. Commonly reported infections included upper-respiratory-tract infections and urinary tract infections. Notably, herpes zoster infections, though infrequent, were more prevalent in the tofacitinib group than in the placebo group. Moreover, tofacitinib raised concerns regarding malignancy risk. A European study by Bezzio et al. (2021) reported a higher rate of cancer events in IBD patients treated with JAK inhibitors, predominantly driven by an increased risk of non-melanoma skin cancer. The incidence of malignancies appeared to be dose-dependent, further necessitating cautious risk–benefit assessments in clinical decision making [31].

As the safety profile of tofacitinib was scrutinized, upadacitinib emerged as another JAK inhibitor evaluated in the IBD context. Clinical trials of upadacitinib in ulcerative colitis yielded promising results, with a notable proportion of patients achieving clinical remission and mucosal healing (Sandborn et al., 2021) [32]. Nevertheless, the safety aspects warrant meticulous attention. Infections remained a notable concern, as upper-respiratory-tract infections, nasopharyngitis, and sinusitis were frequently reported in the upadacitinib-treated groups. While the incidence of herpes zoster infections was relatively low, the risk was elevated compared to the placebo group. Additionally, elevations in liver enzyme levels and decreases in hemoglobin concentrations were noted, necessitating regular monitoring. Therefore, upadacitinib, like tofacitinib, is associated with a distinct safety profile in the context of IBD.

The evaluation of JAK inhibitors in the context of IBD extends to filgotinib, which also showed its potential in clinical trials. Filgotinib, in a Phase 2 study by Sandborn Panés et al. (2019), exhibited a higher rate of clinical remission among patients with moderate-to-severe Crohn’s disease compared to the placebo group [32]. The safety analysis pinpointed common adverse events, including nasopharyngitis and headache, akin to the patterns observed with other JAK inhibitors. Intriguingly, the filgotinib trials did not uncover elevated risks of herpes zoster infections or malignancies. This distinction underscores the importance of distinguishing between JAK inhibitors regarding their safety profiles in the context of IBD.

Baricitinib, while primarily indicated for rheumatoid arthritis and not specifically approved for IBD, also finds mention in the context of immune-mediated diseases. Baricitinib’s safety profile becomes relevant when exploring its off-label use in patients with IBD, where it has shown efficacy. An analysis of real-world data conducted by Narula et al. (2018) examined the outcomes of IBD patients treated with baricitinib [33]. While gastrointestinal and herpes zoster infections were reported, the overall safety profile appeared acceptable. However, the dearth of large-scale studies necessitates further exploration of baricitinib’s safety and efficacy in IBD patients.

In conclusion, the introduction of Janus kinase (JAK) inhibitors has enriched the therapeutic options for patients with Inflammatory Bowel Diseases (IBD). The efficacy of these agents in inducing clinical remission and mucosal healing marks a significant advancement. However, it is imperative to recognize the nuances of their safety profiles, especially in the context of IBD. Tofacitinib, upadacitinib, filgotinib, and the off-label use of baricitinib entail distinct safety considerations, with variations in infection risks, malignancy concerns, and adverse events. The decision to employ JAK inhibitors in treating IBD should be guided by a meticulous assessment of a patient’s unique characteristics, the corresponding risk–benefit ratio, and the availability of alternative treatment options. Future research endeavours are vital to refine our understanding of these safety profiles and optimize the care of patients with IBD.

## 4. JAK Inhibitors in Dermatologic Conditions: Safety and Adverse Events

The advent of Janus kinase (JAK) inhibitors has not only transformed the treatment landscape of immune-mediated diseases but also ushered in a new era in the management of dermatologic conditions. In particular, JAK inhibitors have shown promise in the treatment of alopecia areata, atopic dermatitis, vitiligo, psoriasis, and other skin-related disorders. While their efficacy in these conditions has been widely recognized, it is crucial to delve into the safety profiles and adverse events associated with the use of JAK inhibitors in dermatology [34].

### 4.1. Alopecia Areata

Alopecia areata (AA), an autoimmune disorder characterized by hair loss, has been one of the key areas of focus for JAK inhibitor research [34]. In a two-center, open-label, single-arm trial, 66 patients with severe AA, alopecia totalis (AT), or alopecia universalis (AU) were treated with tofacitinib citrate, a JAK inhibitor (Liu et al., 2019) [35,36]. The study reported that 32% of the treated patients experienced a 50% or greater improvement in their Severity of Alopecia Tool (SALT) score. While the results demonstrated the efficacy of tofacitinib in promoting hair regrowth, it was also evident that the response was not durable. After discontinuation of the drug, patients experienced disease relapse in approximately 8.5 weeks.

Adverse events in the study were generally limited to grade I and II infections. However, it is worth noting that the long-term safety of JAK inhibitors in the treatment of AA requires further investigation, particularly in larger patient cohorts and with extended follow-up periods. The transient nature of responses and the necessity for continuous therapy raise important questions regarding the long-term risks and benefits of JAK inhibitors with regard to this specific dermatologic condition.

### 4.2. Atopic Dermatitis

The application of JAK inhibitors in treating atopic dermatitis (AD), a chronic inflammatory skin disease, has also garnered attention. Notably, upadacitinib and abrocitinib have demonstrated efficacy in the treatment of AD in clinical trials, with a notable reduction in pruritus and an improvement in disease severity (Guttman-Yassky et al., 2020). While these findings highlight the potential of JAK inhibitors in managing AD, the corresponding safety aspects must also be considered [37].

Acne was reported as a side effect in some AD patients receiving abrocitinib. Additionally, routine monitoring of lipid levels is advised, as elevations in both low-density lipoprotein (LDL) and high-density lipoprotein (HDL) cholesterol levels were noted in patients treated with abrocitinib and deucravacitinib, two other JAK inhibitors (Lee et al., 2022) [38]. These observations emphasize the importance of continuous monitoring to assess potential cardiovascular risks among patients undergoing long-term treatment with JAK inhibitors for AD.

### 4.3. Vitiligo

Vitiligo, a chronic depigmenting disorder of the skin, has been the focus of clinical trials assessing the safety and efficacy of JAK inhibitors. Ruxolitinib, a JAK inhibitor, has been evaluated for its effectiveness in treating vitiligo (Harris et al., 2016) [39]. While the corresponding study reported favourable outcomes in terms of repigmentation, it is crucial to consider the safety implications.

The application of topical ruxolitinib resulted in localized adverse events, including application-site acne and pruritus. Nevertheless, these events were relatively rare and manageable. The cited study did not uncover notable risks of systemic adverse events, malignancies, or severe infections. The safety profile of JAK inhibitors for vitiligo suggests a favourable risk–benefit ratio, with localized adverse events being outweighed by the potential for repigmentation, which holds significant promise for improving the quality of life of vitiligo patients [40,41].

### 4.4. Psoriasis

Psoriasis, a chronic inflammatory skin disorder, has witnessed the emergence of deucravacitinib, a JAK inhibitor, as a potential treatment option. Clinical trials have reported promising results in terms of improving disease severity, with low rates of major cardiovascular events and venous thromboembolism (Bissonnette et al., 2021). However, as with other dermatologic conditions, the safety aspects warrant consideration [42].

Nausea and diarrhoea were among the common gastrointestinal side effects observed for deucravacitinib-treated patients. These events were generally mild to moderate in severity and manageable. The corresponding study highlighted the importance of assessing the long-term safety and monitoring potential risks associated with deucravacitinib, particularly in the context of psoriasis patients, some of whom may have other comorbidities, including cardiovascular risk factors [43,44].

The introduction of JAK inhibitors has ushered in a new era in the treatment of various dermatologic conditions, offering hope to patients who have often struggled with limited therapeutic options. While the efficacy of these agents in treating conditions such as alopecia areata, atopic dermatitis, vitiligo, and psoriasis is promising, the safety profiles are subject to scrutiny. Adverse events, while generally manageable, underscore the need for ongoing monitoring, especially when considering long-term treatment. These dermatologic conditions present distinct challenges and considerations, and further research is necessary to optimize the risk–benefit assessment and long-term management of patients.

## 5. JAK Inhibitors and Venous Thromboembolism (VTE)

**Figure 4:**
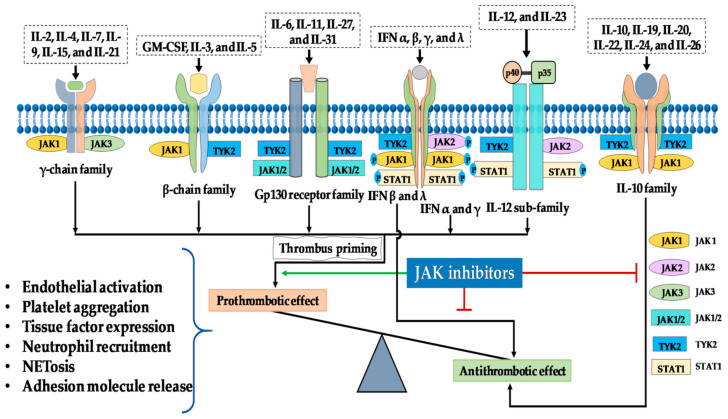
Possible mechanism behind the prothrombotic effects associated with the administration of Jakinibs. JAKs in various combinations bind to cytokine receptors that transmit prothrombotic and proinflammatory signals from a wide range of cytokines. With the exception of IL-10, IFNβ, and IFNλ that have anti-thrombotic potential, signalling downstream of these cytokines creates a permissive background for thrombus formation. Non-specific Jakinibs that target both of the IL-10R-associated JAKs (JAK1 and TYK2) or IFNβ- and IFNλ-associated JAKs (JAK1 and TYK2) may result in an imbalance in the pro- and anti-thrombotic signalling resulting in thrombus priming [47].

The relationship between Janus kinase (JAK) inhibitors and venous thromboembolism (VTE) is a topic of significant concern and scrutiny (Figure 4). VTE, which encompasses deep vein thrombosis (DVT) and pulmonary embolism (PE), poses a substantial health risk, and understanding its association with JAK inhibitors is crucial for the safe and effective treatment of various immune-mediated diseases [45,46].

### 5.1. Tofacitinib in Focus

Tofacitinib, one of the earliest JAK inhibitors to gain approval for immune-mediated diseases, has been extensively studied. Clinical trials, such as the Oral Rheumatoid Arthritis Trials (ORAL) program, revealed an increased risk of VTE events in tofacitinib-treated patients (Cohen et al., 2014) [7]. However, subsequent analyses have provided a more nuanced perspective. Different studies noted that in the general tofacitinib rheumatoid arthritis (RA) population, the risk of VTE was not significantly higher than that associated with tumour necrosis factor inhibitors (TNFis) [48,49]. This apparent disparity in findings underscores the complexity of assessing VTE risk.

### 5.2. Upadacitinib and Filgotinib: Further Insights

Upadacitinib and filgotinib, two newer entrants in the JAK inhibitor landscape, have also undergone scrutiny. In the SELECT program, which investigated upadacitinib in patients with rheumatoid arthritis, the incidence of VTE was found to be relatively low (Fleischmann et al., 2019). While this suggests a favourable safety profile, a comprehensive evaluation across multiple studies is required to validate these findings [15].

Filgotinib, evaluated in the FINCH clinical trial program, reported VTE events at a rate that was consistent with that of the general RA population, similar to the observations with tofacitinib (Combe et al., 2021). The intricate interplay of factors contributing to VTE risk is evident in these varying results [50].

### 5.3. Baricitinib: A Tale of Risk Mitigation

Baricitinib, another well-established JAK inhibitor, has demonstrated some interesting risk patterns. The RA-BEGIN study found that the risk of PE was higher in patients taking a higher dose of baricitinib (4 mg daily) than in those taking a lower dose (2 mg daily) (Dougados et al., 2017). This dose-dependent relationship emphasizes the importance of dosing considerations in JAK inhibitor therapy [27].

Notably, baricitinib’s journey also involved successful efforts to mitigate VTE risk. Subsequent research revealed that the risk of VTE was not significantly different from that associated with TNFis thanks to dose adjustments and risk minimisation strategies. The proactive measures taken to optimize safety profiles underscore the capacity to fine-tune the risk–benefit balance using JAK inhibitors [27,28].

## 6. Thromboembolic Events and JAK Inhibitors in Various Immune-Mediated Diseases

The potential link between thromboembolic events (TE) and Janus kinase (JAK) inhibitors has garnered significant attention in the realm of immune-mediated diseases. As JAK inhibitors have become established therapies for a range of conditions, understanding the incidence and clinical implications of TE in various immune-mediated diseases has become increasingly important [21,51,52,53,54,55].

### 6.1. Rheumatoid Arthritis (RA): The Pioneering Ground

Rheumatoid arthritis (RA) has served as a pioneering field for investigating the risk of TE associated with JAK inhibitors. Tofacitinib, the first JAK inhibitor approved for RA treatment, was at the forefront of these explorations. Initial concerns arose from observations in clinical trials, particularly the Oral Rheumatoid Arthritis Trials (ORAL) program. In this program, the risk of major adverse cardiovascular events (MACE) and deep vein thrombosis (DVT) was reportedly higher with tofacitinib (Cohen et al., 2014) [7,21]. This raised essential questions about the overall cardiovascular and thrombotic safety of JAK inhibitors.

### 6.2. Navigating the Risk Landscape

Subsequent studies navigated the risk landscape more comprehensively. Kremer et al. (2021) examined data from the Consortium of Rheumatology Researchers of North America (CORRONA) registry, further elucidating the cardiovascular safety profile of tofacitinib [20]. They found that the rates of MACE and VTE were not significantly different from those associated with tumour necrosis factor inhibitors (TNFis) within the general RA population. This highlights the evolving understanding of JAK inhibitor-associated TE risk and the need to consider a broader context of cardiovascular safety.

### 6.3. Evolving JAK Inhibitors: Insights from Upadacitinib and Filgotinib

Upadacitinib and filgotinib, newer entrants to the JAK inhibitor landscape, have brought fresh perspectives. In the SELECT program, which evaluated upadacitinib in RA patients, the incidence of TE was relatively low (Fleischmann et al., 2019). While this hints at a favourable safety profile, the complexity of TE risk assessment in the context of immune-mediated diseases requires continuous scrutiny [5,23].

Filgotinib, as assessed in the FINCH clinical trial program, provided insights into the thrombotic risk landscape [24]. Notably, the overall TE risk appeared consistent with that observed in the general RA population, akin to the observations with tofacitinib (Combe et al., 2021) [50]. This reinforces the idea that interpreting TE risk requires a nuanced understanding of the interplay between the specific JAK inhibitor, the underlying disease, and individual patient factors.

### 6.4. Inflammatory Bowel Diseases (IBD): Broader Applications

Beyond RA, JAK inhibitors have found applications in inflammatory bowel diseases (IBD). In the context of IBD, the JAK inhibitor tofacitinib was evaluated for its safety and efficacy. The OCTAVE clinical trial program focused on tofacitinib in patients with ulcerative colitis, providing some insights into TE risk (Sandborn et al., 2017) [30]. Importantly, the rates of DVT and PE were found to be comparable to those for patients treated with a placebo. These findings underscore that the specific immune-mediated disease being treated plays a crucial role in determining TE risk.

### 6.5. Influence of Age, Comorbidities, and Dosages on Safety of JAK Inhibitors

**Figure 5:**
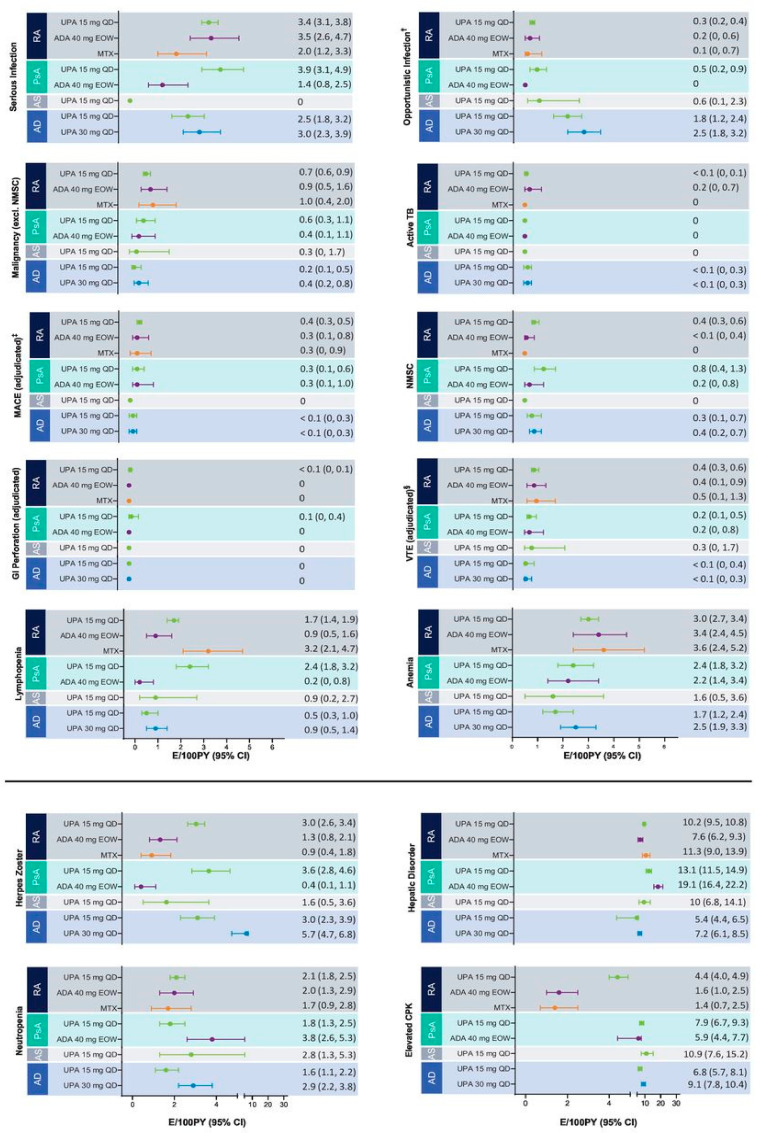
Exposure-adjusted event rates for TEAEs of special interest [56]. † excluding TB, oral candidiasis, and herpes zoster. ‡ defined as cardiovascular death, non-fatal myocardial infarction, and non-fatal stroke. § including deep vein thrombosis and pulmonary embolism. RA: UPA 15 mg QD (*n* = 3209), ADA 40 mg EOW (*n* = 579), MTX (*n* = 314); PsA: UPA 15 mg QD (*n* = 907), ADA 40 mg EOW (*n* = 429); AS: UPA 15 mg QD (*n* = 182); AD: UPA 15 mg QD (*n* = 1340), UPA 30 mg QD (*n* = 1353). AD, atopic dermatitis; ADA, adalimumab; AS, ankylosing spondylitis; CPK, creatine phosphokinase; E, event; EOW, every other week; GI, gastrointestinal; MACE, major adverse cardiovascular event; MTX, methotrexate; NMSC, non-melanoma skin cancer; PsA, psoriatic arthritis; PY, patient years; QD, once a day; RA, rheumatoid arthritis; TB, tuberculosis; TEAE, treatment-emergent adverse event; UPA, upadacitinib; VTE, venous thromboembolic event.

A safety analysis of upadacitinib, involving 6991 patients with a maximum follow-up of 5.45 years, revealed a generally consistent safety profile across cases of rheumatoid arthritis (RA), psoriatic arthritis (PsA), ankylosing spondylitis (AS), and atopic dermatitis (AD). Notably, variations in adverse events (AEs) were observed, likely influenced by factors such as age, comorbidities, and dosages (Figure 5) [56].

In terms of dosages, the analysis indicated that 30 mg of upadacitinib was associated with an overall increased risk of treatment-emergent adverse events (TEAEs) compared to 15 mg, particularly with respect to AD. The observed differences in TEAEs underscore the importance of considering dosage when evaluating the safety profile of upadacitinib, similar studies are available for the other JAK inhibitors.

Age was a significant factor influencing safety outcomes. For instance, numerically higher rates of malignancy were noted with upadacitinib at 30 mg in treating AD, but the majority of these events occurred within six months after initiating treatment. This temporal aspect suggests the need for careful consideration of age-related factors when assessing the risk of malignancies associated with upadacitinib.

Comorbidities, including baseline corticosteroid use, varied across diseases. Patients with RA had higher levels of baseline corticosteroid use, reflecting differences in treatment recommendations. However, the relationship between corticosteroid use and major adverse cardiovascular events (MACE) or venous thromboembolism (VTE) was not explored. The analysis emphasized the challenge of separating corticosteroid effects from other risk factors, indicating a need for further investigation into these associations.

### 6.6. Understanding the Complex Risk Factors

Understanding TE risk in the context of JAK inhibitors involves unravelling a complex web of risk factors. Some of these include the specific JAK inhibitor used, the dosage, the patient population, and the disease itself. The interaction between these factors creates a multifaceted landscape that requires individualized risk assessments.

The safety of Janus kinase (JAK) inhibitors in the treatment of various immune-mediated diseases is a topic of paramount importance in the field of medicine. These inhibitors have emerged as a promising class of medications, revolutionising the therapeutic landscape for conditions such as rheumatoid arthritis (RA), inflammatory bowel diseases (IBD), and dermatologic conditions. While their efficacy in managing immune-mediated diseases is well-documented, concerns surrounding their safety profile persist. This discussion explores the multifaceted dimensions of JAK inhibitors, with a focus on their safety, informed by a comprehensive review of relevant studies and trials.

## 7. Discussion

The introduction of JAK inhibitors brought about a paradigm shift in the management of immune-mediated diseases. Traditionally, treatments ranged from corticosteroids to disease-modifying antirheumatic drugs (DMARDs) and biologics. However, JAK inhibitors offered new hope to patients by targeting the Janus kinase pathways, which play a pivotal role in immune responses. These medications, including tofacitinib, upadacitinib, filgotinib, and baricitinib, have demonstrated remarkable efficacy in ameliorating disease symptoms and improving quality of life for countless individuals. Patients with RA, IBD, dermatologic conditions, and other immune-mediated diseases have reaped the benefits of these therapies, including pain relief, enhanced mobility, and disease remission. Nevertheless, the use of JAK inhibitors raises legitimate concerns about adverse events, necessitating a comprehensive evaluation of their safety profile.

In the realm of rheumatoid arthritis, a substantial body of evidence supports the effectiveness of JAK inhibitors, particularly tofacitinib, upadacitinib, and baricitinib. These medications have been shown to significantly improve RA management, with noteworthy outcomes related to the American College of Rheumatology criteria and Health Assessment Questionnaire-Disability Index scores. Patients have experienced tangible relief from the burden of RA, which is a debilitating condition characterized by joint pain, inflammation, and progressive damage. However, safety assessments reveal that specific dosages of JAK inhibitors are associated with a higher relative risk of adverse events. In particular, upadacitinib at a dose of 30 mg taken daily, upadacitinib at a dose of 15 mg taken daily, and baricitinib at dose of 4 mg taken daily exhibited elevated risks. Additionally, the risk of infection, notably with herpes zoster, varies among these inhibitors, with tofacitinib at 10 mg twice daily carrying the highest relative risk. This highlights the importance of tailoring the choice of JAK inhibitor and dosage to individual patient characteristics and risk factors.

The application of JAK inhibitors extends beyond RA into the domain of inflammatory bowel diseases. Patients grappling with conditions like Crohn’s disease and ulcerative colitis have witnessed the positive impact of these medications on disease management. Notably, tofacitinib has proven effective in treating ulcerative colitis, offering relief from the debilitating symptoms that these patients face, such as abdominal pain and diarrhoea. However, the corresponding safety assessment indicates that tofacitinib has a concerning association with venous thromboembolic events (VTEs), potentially leading to deep vein thrombosis and pulmonary embolism. This underscores the importance of vigilant monitoring and developing individualized treatment plans to mitigate this risk. Additionally, it is essential to note that the diseases themselves may contribute to the risk of VTE, and understanding this interaction is crucial in clinical decision making.

JAK inhibitors have also found application in the management of dermatologic conditions, including alopecia areata, psoriasis, vitiligo, and atopic dermatitis. These conditions can have a profound impact on a patient’s psychological well-being and overall quality of life due to visible skin and hair manifestations. JAK inhibitors, such as tofacitinib, offer a new avenue of treatment, showcasing promising outcomes in promoting hair regrowth and alleviating skin symptoms. Nevertheless, this discussion highlights the need to consider specific adverse events associated with dermatologic conditions, such as application site reactions, when utilising topical JAK inhibitors.

## 8. Conclusions

The emergence of JAK inhibitors has significantly reshaped the therapeutic landscape for immune-mediated diseases, presenting substantial benefits in symptom management and enhanced quality of life for patients. However, a comprehensive understanding of their safety profile is imperative. The combined insights from the reviewed studies highlight the efficacy of JAK inhibitors in diverse conditions such as rheumatoid arthritis, inflammatory bowel diseases, and dermatologic conditions.

Adverse events associated with JAK inhibitors exhibit variability, with infections and malignancies more prevalent at certain dosages. The importance of personalized treatment decisions, meticulous patient selection, and continuous monitoring cannot be overstated. The heightened risk of venous thromboembolism (VTE), notably linked to specific JAK inhibitors, underscores the need for diligent risk assessment and ongoing surveillance.

JAK inhibitors exhibit promise in the realm of dermatologic conditions but prompt concerns regarding the risk of herpes zoster infections. The elevated VTE risk for patients with immune-mediated diseases further emphasizes the need for careful consideration of the overall risk profile.

In summary, making informed, individualized treatment decisions and conducting baseline risk factor assessments are critical aspects of employing JAK inhibitors for treating immune-mediated diseases. As research progresses, our understanding of the safety profile will undoubtedly deepen, facilitating treatment optimisation while mitigating potential risks. Continuous exploration and monitoring will be pivotal in refining the management approach for immune-mediated diseases in the era of JAK inhibitors.

## Figures and Tables

**Table 1:** Commercially available JAK inhibitors in Europe.

Trade Names	Janus Kinase Inhibitors
Xeljanz (Pfizer)	Tofacitinib
Rinvoq (Abbvie)	Upadacitinib
Olumiant (Elli Lilly)	Baricitinib
Cibinqo (Pfizer)	Abrocitinib
Jyseleca (Gilead)	Filgotinib

**Table 2:** Available clinical trials on efficacy and safety of JAK inhibitors (PubMed).

Authors	Title of Article/Study
J. Tesser et al.	Efficacy and safety of tofacitinib in patients with rheumatoid arthritis by previous treatment: post hoc analysis of phase II/III trials [9]
X. Liao et al.	Efficacy and safety of different Janus kinase inhibitors combined with methotrexate for the treatment of rheumatoid arthritis: a single-center randomized trial [10].
A.V. Ramanan et al.	Baricitinib in juvenile idiopathic arthritis: an international, phase 3, randomised, double-blind, placebo-controlled, withdrawal, efficacy, and safety trial [11]
C.T. Deakin et al.	Comparative Effectiveness of Adalimumab vs. Tofacitinib in Patients With Rheumatoid Arthritis in Australia [12].
M.M. Khan et al.	Tofacitinib versus methotrexate as the first-line disease-modifying antirheumatic drugs in the treatment of rheumatoid arthritis: An open-label randomized controlled trial [13]
M.Q. Mao et al.	The evaluation of JAK inhibitors on effect and safety in alopecia areata: a systematic review and meta-analysis of 2018 patients [14].
R. Fleischmann et al.	Safety profile of upadacitinib in patients at risk of cardiovascular disease: integrated post hoc analysis of the SELECT phase III rheumatoid arthritis clinical programme [15]
P. Eriksson et al.	Clinical experience and safety of Janus kinase inhibitors in giant cell arteritis: a retrospective case series from Sweden [16].
X. Tong et al.	Cardiovascular risk in rheumatoid arthritis patients treated with targeted synthetic and biological disease-modifying antirheumatic drugs: A multi-centre cohort study [17]
M. Russell et al.	JAK inhibitors and the risk of malignancy: a meta-analysis across disease indications [18].

## Data Availability

Not applicable.
